# Young people's lived experience expertise: Insights from the DigiCAT project to develop a counterfactual analysis tool for mental health data

**DOI:** 10.1002/jcv2.70033

**Published:** 2025-07-26

**Authors:** Marie Allitt, Helen Wright, Aja Murray, Ingrid Obsuth

**Affiliations:** ^1^ Department of English and Scottish Literature School of Literatures, Languages, and Cultures University of Edinburgh Edinburgh UK; ^2^ Department of Psychology School of Philosophy, Psychology and Language Sciences University of Edinburgh Edinburgh UK; ^3^ Population Health Sciences Institute, Medical Sciences Faculty Newcastle University Newcastle upon Tyne UK; ^4^ Department of Clinical & Health Psychology School of Health in Social Science University of Edinburgh Edinburgh UK

**Keywords:** adolescence, counterfactual analysis, digital tool, lived experience, mental health

## Abstract

**Background:**

Lived experience (LE) expertise is increasingly recognised as a vital component in mental health research. In our project to develop a digital tool for counterfactual analysis (DigiCAT), with an emphasis on researching active ingredients for adolescent mental health, we incorporated LE expertise across the lifecycle of tool development and dissemination.

**Methods:**

We consulted young person advisory groups (YPAGs; aged 11–18 years old) across three project phases—Discovery, Prototyping, and Dissemination—using structured discussions, ranking exercises, and iterative feedback loops to shape research priorities, tool design and user tutorials, and dissemination strategies of the tool.

**Results:**

The YPAGs advised on active ingredients and features of such ingredients that existing research has not taken into account. Examples include young person advisory group (YPAG) suggestions to prioritise research in social media, peer relationships, and teacher‐student relationships. We incorporated these suggestions as illustrative examples in our tutorial paper introducing DigiCAT to our target audience, to demonstrate how insights from YPAGs can inform the use of the tool in research, by guiding areas of study. These areas were also prioritised in our own empirical analyses using the tool. Additionally, the YPAG contributed practical guidance on how to effectively involve youth LE experts in both research and digital tool development processes.

**Conclusions:**

The integration of LE expertise fundamentally shaped the development of our tutorial paper, influencing both its instructional components and its broader discussion of applications in mental health research. This project highlights the value of embedding LE perspectives into research guidance for software use, offering a model for future mental health and digital innovation initiatives.

## Key Points

- What's known: lived experience (LE) expertise is increasingly valued in research, but incorporating it meaningfully, particularly in digital innovation projects, remains a challenge.
- What's new: This article presents a case study that addresses the gap in the literature by integrating LE expertise into the development of a digital tool, a practice with little existing guidance.
- What's relevant: The insights offered are highly relevant for policy and service development in mental health. They demonstrate how LE expertise can be systematically incorporated into research priorities, tutorial development, and dissemination strategies, even when the end users are not LE advisors themselves.
- Further implications: This work has particular significance for involving young people in mental health research and shaping ethical practices around the use of LE expertise in future digital tool development and research initiatives.

## Graphical abstract

In our project to develop a digital tool for counterfactual analysis, with an emphasis on researching active ingredients for adolescent mental health, we incorporated lived experience expertise across the lifecycle of tool development and dissemination. We consulted with young person's advisory groups, where discussion shaped our research questions, guided the direction of the tool, and informed routes for dissemination.

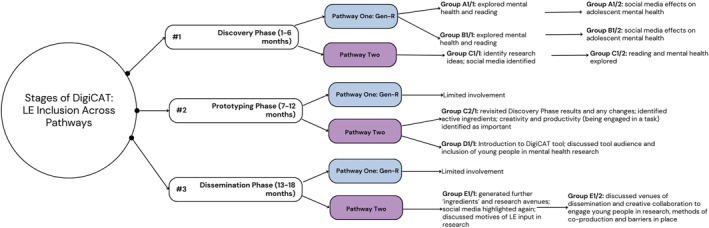

## INTRODUCTION

Adolescence is a critical period for mental health, with up to one in seven adolescents worldwide experiencing mental health difficulties (World Health Organization, 2024). Despite growing awareness of these challenges, there remains an urgent need for research approaches that identify effective interventions and ensure they are relevant to young people's lives. One promising strategy is the integration of lived experience (LE) expertise in adolescent mental health research to improve relevance, accessibility, and impact. This paper presents an example of how LE expertise can inform research priorities, tutorial development, and dissemination strategies in the context of digital tool development for mental health research.

The inclusion of LE input in research has gained increasing recognition as a vital component in ensuring relevance, accessibility, and effectiveness, particularly in fields like mental health (Rose & Kalathil, 2019). However, the danger of such inclusion becoming tokenistic or reduced to a box‐ticking exercise remains a persistent concern. This is particularly true in areas where appropriate methodologies for meaningful LE inclusion are not yet well‐established, such as in digital tool design for mental health research. Integrating LE expertise can pose challenges, for example, in balancing power dynamics, ensuring genuine collaboration, and overcoming resource constraints (Madden & Speed, 2017). In this paper, we address these issues by illustrating the process and benefits of incorporating adolescent LE within a software development research project. Specifically, we explore the role and the limits of young person advisory groups (YPAGs) in shaping the design of a digital tool for counterfactual analysis (DigiCAT), highlighting both the challenges and solutions encountered. Young people were involved in this project as advisors, rather than as research participants, or co‐producers. Their contribution was consultative: they provided insights and guidance grounded in their LE to inform specific aspects of the project, such as shaping research priorities, informing tutorial examples, and advising on dissemination strategies. They did not hold decision‐making power or participate in the co‐design or co‐development of the digital tool itself. This distinction helped ensure their involvement was meaningful and appropriate to the aims and scope of the project.

Adolescence represents a critical period marked by developing and changing identities (Blakemore & Mills, 2014), bringing about rapid changes and increased susceptibility to novel risk factors, including but not limited to physical transformations, peer relationships, academic pressures, and sexual exploration (Ogden & Hagen, 2018; Viner Russell et al., 2015). Numerous longitudinal studies have demonstrated an increase in the prevalence of mental disorders from childhood to adolescence (e.g., Costello et al., 2003). Adolescent mental health issues appear to be increasing (a trend which began even before the pandemic), with rising rates of anxiety, depression, and suicides (Cybulski, et al., 2021).

Although adolescence is a period in which we observe increasing development in agency and autonomy, this change has yet to be fully reflected in how adolescents are involved in research (Bettis et al., 2023). To uncover aspects of adolescent mental health that extend beyond current adult‐centric models, meaningful engagement, such as incorporating LE expert input of adolescents, is needed and can significantly enhance our understanding of these issues (Twivy et al., 2023). Research recognises the value of LE inclusion, highlighting its role in promoting mental health awareness, reducing stigma, and advocating for systemic change (Al‐Busaidi, 2008; Patel et al., 2018). Engaging young people as advisors provides researchers with a better understanding of the experiences, needs, and preferences of this demographic, leading to, for example, more effective and youth‐friendly interventions and support services (McCabe et al., 2023). It can also serve to enhance their skill and knowledge and empower young people and to humanise the research process, putting ‘faces to the data’ (Honey et al., 2020; Watson et al., 2023).

Despite the recognised value of LE expertise in research, there are notable challenges in its implementation. For example, highly technical or specialised research projects may not easily lend themselves to LE involvement because they might require the provision of substantial training for expert advisors to engage meaningfully (e.g., Di Lorito et al., 2020). Furthermore, while best practice guidelines for involving LE experts offer general advice and principles, they often lack specific guidance on how to involve such expertise (Hawke et al., 2025).

Our illustrative example of these challenges is the DigiCAT project, which focused on the development of a digital tool to implement complex statistical analyses of mental health data. Target users are mental health researchers. Thus, while researchers are directly involved in using the tool, adolescent LE experts may be impacted by the research outputs in less direct but significant ways. For example, a user of the tool may identify a novel intervention target, which could potentially lead to the development of a new treatment for young people affected by mental health issues. In such cases, adolescent LE advisors are crucial because their insights and feedback can provide a deeper understanding of the specific needs, preferences, and challenges faced by the group they represent.

This paper offers an example of integrating LE in mental health research effectively across the lifecycle of digital tool development and dissemination, as well as a discussion of the benefits and challenges of such methods. By embedding the perspectives of YPAGs into the design and refinement of DigiCAT, we highlight the importance of tailoring research methodologies to meaningfully include adolescent voices. Our approach illustrates how YPAG involvement can identify and address gaps in current research, foster innovations in digital tool functionality, and ensure the principled use of counterfactual analysis in mental health studies. In doing so, we provide a model for future projects seeking to balance academic rigour with the real‐world relevance gained through LE expertise.

## LE EXPERT INVOLVEMENT APPROACH

### Context

‘DigiCAT’ is a digital tool development project in which we created a tool to promote the uptake and principled use of counterfactual analysis of mental health data to help accelerate progress in identifying and prioritising mental health intervention targets (Murray et al., 2024). Counterfactual analysis is a method that evaluates ‘what if’ scenarios by estimating the outcomes that might have occurred under different conditions. Using propensity score methods, the tool accounts for confounding variables either by matching individuals with similar characteristics, or by weighting them, thus approximating the conditions of a randomised controlled trial in observational data (Austin, 2011).

Traditionally, these advanced methods require access to programming‐heavy platforms or expensive licenced software, posing significant barriers for researchers or practitioners without specialised skills or resources. The tool offers a user‐friendly, point‐and‐click interface that guides users through the steps of propensity score analysis. This includes selecting variables, assessing balance, and interpreting results, all within a visually intuitive platform.

The purpose of DigiCAT is thus twofold: first, to advance understanding of the nuanced relationships between active ingredients (e.g., modifiable risk factors) and adolescent mental health; and second, to bridge the gap between statistical complexity and practical application by developing a user‐friendly digital tool. Further information on the tool can be found at: DigiCAT Tutorials (uoe‐digicat.github.io).

DigiCAT was developed over the course of three project phases: the Discovery Phase, Prototyping Phase, and Dissemination Phase, all funded by the Wellcome Trust. A Lived Experience team, led by a Lived Experience Lead, adhering to best practice guidance (ALLIANCE, 2022; Hawke et al., 2022; Honey et al., 2020) was formed to ensure meaningful LE involvement throughout the project. LE engagement occurred through a series of YPAGs, whose insights were shared across the broader team, including the tool development and dissemination teams, informing various aspects of the project—from tutorial guidance to dissemination strategies and academic research outputs. Similarly, other sub‐teams contributed to the content of LE consultations and members of the tool development team actively participated in designing and co‐leading selected LE engagement sessions. The involvement of YPAGs was critical to ensuring that the tool's development and its broader implications were aligned with the lived realities and priorities of young people. Importantly, the funder supported meaningful LE involvement not only by enabling it to be appropriately costed into the project budget but also by facilitating a series of learning sessions on LE involvement, which helped build the capacity of participating teams to engage effectively with LE experts.

## DEVELOPING COLLABORATIONS WITH YP

### Strategy

Research (e.g., Mandoh et al., 2023) has shown that YPAGs can often be unrepresentative, and often made up of young people with links to clinicians and researchers. We therefore implemented several measures to maximise the chances that our groups were diverse, by recruiting through multiple channels. We developed two pathways with our YPAGs: the first used an established YPAG, Bristol's Generation R (Gen‐R), and the second involved the recruitment of a tailored group specifically for this project. The Gen‐R YPAG, pathway one, which operates with an open application process, was originally formed through engagement with schools, youth groups, and charities to recruit broadly. Young people can also apply to join via the ARC West's website and many also join via word of mouth from current members. Pathway two recruited by advertisements via a range of mailing groups and social media platforms.

Rather than monitor diversity, we prioritised sensitivity and equity in our approach, treating young people as collaborators rather than collecting personal information such as gender and ethnicity. We discuss the limitations of this approach further in our discussion. All YPAG sessions were conducted online, with the aim of maximising geographical inclusivity and accessibility for individuals from diverse locations to contribute, including the majority from Scotland and England, one advisor from Ireland, two advisors from Romania, and two advisors from France.

Gen‐R advisors (Pathway One) were divided into two age groups: 11–13 and 14–17 years, reflecting developmental differences in adolescence. According to Piaget, children aged 11–13 transition into the formal operational stage, where abstract reasoning emerges, while those aged 14–17 develop stronger critical thinking skills. Socially, younger adolescents are more peer‐influenced and self‐conscious, whereas older ones focus on identity and independence (Steinberg, 2017).

The 11–13 age group experiences puberty, increasing social awareness, and evolving identity, self‐esteem, and peer relationships (Blakemore & Mills, 2014). In contrast, 14–17‐year‐olds develop advanced problem‐solving, moral reasoning, and future‐oriented thinking, alongside stronger peer influences and greater independence (Steinberg, 2005; Steinberg & Morris, 2001).

These differences impact how each group experiences mental health, informing our YPAG consultations. Separating them allowed facilitators to tailor discussions to their cognitive and social needs, ensure balanced participation, and address age‐specific concerns, such as school transitions for younger advisors and future planning for older ones. Advisors affirmed that different ages face distinct stresses, reinforcing our approach.

**FIGURE 1:**
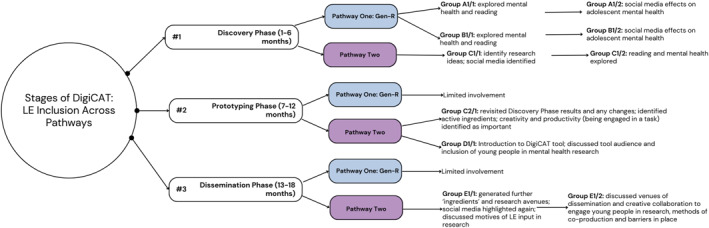
LE involvement and YPAG structures across DigiCAT funding phases. DigiCAT, digital tool for counterfactual analysis; LE, lived experience; YPAG, young person advisory group.

Pathway two YPAGs had less age variation in the first two phases, so we held these together (11–15) but split them in the third phase (12–14) and (15–18) as new members joined (see Figure 1). Some but not all advisors continued their involvement throughout the phases while others were added later on, reflecting a need to be flexible about young people's involvement as advisors.

### Inclusion criteria

To participate in organised advisory groups, adolescents needed to be between 11 and 18 years old. Our LE experts were individuals who had direct, first‐hand LEs of being an adolescent at the time of study, and potentially experiences navigating mental health challenges during this period, and/or had a friend navigating these. There was no requirement for young people to possess or disclose any specific mental health diagnoses to take part in the study. While advisors were not explicitly asked to disclose personal mental health experiences, they frequently discussed factors such as academic pressures, social relationships, and social media use—areas known to intersect with adolescent mental health—enabling capture of a wide range of perspectives, including moments of stress, anxiety, or low mood, as well as broader insights into the influences on adolescent wellbeing without requiring advisors to self‐identify as having specific mental health conditions.

While there were no additional explicit inclusion criteria, it is important to note that the necessity to participate in online group discussions at specific times may have implicitly excluded individuals experiencing significant social anxiety, communication difficulties, or mental health issues.

### Compensation

Although LE advisors often volunteer their time, our project included budget provisions to compensate advisors with vouchers (£30 vouchers per 60‐min YPAG session).

## STRUCTURE

The overall flow of the YPAGs across the project and content of the consultations at different stages are summarised in Figure 1.

### Discovery phase

This initial phase focused on identifying and prioritising research questions related to adolescent mental health. We conducted three YPAG sessions where advisors engaged in a ranking exercise of five candidate ‘active ingredients’ (factors affecting mental health) including sleep, engaging with the arts (e.g., music, acting, reading), social media, supportive relationships with teachers, and physical exercise. Advisors independently ranked these factors, followed by group discussions to share their rankings and reflect on their choices. These discussions facilitated collective insights and provided critical feedback, with social media and reading emerging as particularly relevant areas for further exploration.

Although no formal qualitative analysis was conducted, we identified recurring patterns and notable divergences in the rankings. Transcripts of the discussions were reviewed and compared to contextualise these findings within the broader literature on adolescent mental health. This ensured that the perspectives of the advisors were meaningfully integrated into the tool's development and informed subsequent phases of the project.

Between sessions, we conducted inverse‐probability treatment weighted analyses within DigiCAT, which assessed the impact of social media use on wellbeing and depression scales using the Millennium Cohort Study longitudinal dataset (Centre for Longitudinal Studies, 2024). During further sessions, advisors interpreted findings, discussed implications, and identified gaps and limitations. The Discovery Phase comprised six 60‐min online sessions, with an average of five advisors per session. Due to resource constraints, Gen‐R groups (Pathway One) were primarily engaged in this phase.

The Discovery Phase shaped the research questions and guided aspects of the development of the tool. The active ingredient ranking exercise refined our focus by identifying social media as a particularly salient topic, which became central to subsequent analyses. Insights from the YPAGs informed the design of analyses, and their collaborative interpretation of findings ensured that research priorities and tool content aligned closely with young people's LEs. This iterative process also laid the foundation for the tutorial example within the publication that introduces the tool to a user audience, which demonstrates its functionality using social media's impact on mental health as a case study. Reflecting these priorities we also prioritised empirical research on social media and adolescent mental health in our analyses and publications.

### Prototyping phase

This phase engaged with the Pathway Two YPAGs. In the first session, we revisited the active ingredients ranking exercise to gather further insights into areas young people felt were most important for supporting adolescent mental health. Advisors also shared activities they believed had a positive impact on their wellbeing, including arts‐related examples, which enriched our understanding of relevant active ingredients. In the second session, we introduced a new group of advisors aged 11–15. During this session, we explored the principles and benefits of involving LE experts in research and invited their feedback on the prototype's initial functionalities and suggestions for potential improvements.

While we aimed to involve YPAGs in the design and refinement of the DigiCAT prototype, we encountered challenges in facilitating meaningful engagement with the more technical aspects of a statistical analysis tool. These sessions highlighted the complexities of involving young people in decisions around tool design and functionality in a way that feels accessible and impactful for them. Nonetheless, their feedback informed our thinking about how to make the tool's purpose and potential applications clearer and more relevant to young people, and shaped the examples and guidance we developed to demonstrate its use. As such, they did not contribute to decisions about the tool's technical design, functionality, or coding.

### Dissemination phase

The final phase emphasised knowledge sharing and expanded the focus to include advisors aged 12–18, incorporating a mix of returning and new advisors to diversify perspectives. Two YPAG sessions were conducted with pathway two groups. In the first session, advisors repeated the ranking exercise to uncover new examples of active ingredients and explored the value and barriers of youth involvement in research. The second session briefly reintroduced the DigiCAT tool, allowing advisors to provide feedback on its perceived utility and intended target users.

By actively engaging YPAGs across all project stages, we integrated their LEs into as many aspects of the project as was appropriate, including shaping research priorities, tutorial examples, and dissemination strategies.

## INSIGHTS AND FINDINGS

The advisors provided a wealth of invaluable insights that we incorporated into further research, refined tool tutorials, and our dissemination strategy. They identified key factors for adolescent wellbeing, including sleep quality, social media usage, interpersonal relationships, pets, spending time outdoors, and physical activity. They noted the nuanced nature of these factors, such as the varying impact of social media based on individual experiences. These insights significantly informed our subsequent analyses on the associations between social media use and mental health, and between reading and mental health, detailed below. Key impacts are discussed below.

### Peer‐reviewed papers: Social media

One early insight from our ranking exercise was the perceived significant impact of social media on adolescent mental health. Over the course of multiple sessions, we garnered more insight into what aspects of social media were considered positive and therefore helpful, in contrast to the potential harms. Our first YPAG took place in February 2023, and it was evident that the recent pandemic lockdowns and school closures were still on our advisors' minds:

[During lockdowns] people did spend a lot more time on social media, which was fair, but I think we’ve not recovered from that and we’re just spending a lot more time on social media when we have all these other things available.

(Advisor 1)

They began to distinguish between times when social media was directly beneficial to them, namely communicating with their friends when they could not see them face to face, while recognising that being glued to their phone is not good for them:

it can keep you connected, so I think it can actually be a good thing, but it can be bad, you can get addicted and do it too long.

(Advisor 13)

Many preferred socialising with their friends in person, but there was awareness for the limitations to this:

it’s more important to meet up in real life than just send a message.

(Advisor 12)

Limitations on time they can spend with friends might be a particular concern for older adolescents who have more demands on their time with higher study loads, and for whom schoolwork and examinations were a significant source of stress. Insights gained over the sessions that first identified social media use as an important active ingredient and later discussed findings of our empirical analysis were fed into the paper presenting those findings. For example, young people emphasised that *how* social media is used is more important than the time spent on it, which shaped our manuscript's discussion on the limitations of how social media use tends to be measured in research studies.

As our young advisors highlighted, social media is not a single, uniform entity but a dynamic, constantly evolving landscape with both positive and negative aspects. This complexity suggests the need to subdivide social media in future studies to assess the influences of different features, such as various applications, form of socialising, familiarity of respondents, and audience size. Including LE expertise is crucial for uncovering and articulating these nuances.

### Peer reviewed papers: Reading and mental health

In contrast to our social media discussions, the importance of reading varied substantially between YPAGs. We approached this project with a working hypothesis about the relationship between reading for pleasure and mental health, which we pursued through the specially recruited YPAG pathway (referred to here as the ‘reading YPAG’) that explicitly focused on reading. As well as researchers engaged with adolescent psychology, our team also included researchers from literary studies and creative writing who were especially invested in following up on this area, building upon existing research. This line of enquiry helped us to situate reading and the wider arts and cultural activities within factors influencing mental health, including prompts to consider the impact of creativity. The reading YPAG pathway highlighted its benefits, describing the sense of calm and focus it offered them:

You’re in your own world and like, you’re the only one that can hear it. It’s not like a movie where everyone can hear it, it’s like your world and you.

(Advisor 9)

It’s kind of like a calming place.

(Advisor 9)

I feel like there is nothing else, just the book and me.

(Advisor 7)

You can use all your focus.

(Advisor 5)

reading is something you can enjoy and it takes the stress out of you.

(Advisor 5)

They identified this sense of calm as a mechanism contributing to positive mental wellbeing. In the other, non‐reading specific, YPAG pathway, the ranking exercise indicated that reading was not seen as an important ingredient for mental health. This discrepancy suggests that involving different YPAGs can uncover a wider range of perspectives, highlighting the variability in how different factors impact mental health. It indicates that reading's impact may be more pronounced in individuals who already have a strong preference for it and likely reflects the fact that we specifically advertised for LE advisors for a project that would look at reading and mental health.

The preliminary analyses of our empirical paper were mixed, overall showing no substantial or robust evidence for the relation between reading and mental health. When asked about this, the reading YPAG members stated:

it’s quite confusing for me because I think I am happier reading than I was when I didn’t read.

(Advisor 8)

When asked for their interpretation of the findings, advisors mentioned that the genre of a book makes a difference, a nuance not captured in the survey measure used for reading in our analyses:

I mean, some books have a calmer tone to them, whereas some are more action‐packed. So, it does just depend on the part you’re reading in the book.

(Advisor 8)

some books can be more influential than others to different people, so it doesn’t matter how much you read. It matters what you read.

(Advisor 8)

it’s really about what you read, but I think it can really help your mental health.

(Advisor 8)

Notably, reading YPAG members also highlighted the impact of age on reading, and relatedly the impact of school‐related instructional reading on enjoyment and engagement:

I remember when I was quite young, I actually despised reading because I have a spelling disability and I would get really annoyed when teachers and adults would tell me that you just have to do it ‘cause it’s a life skill and they always said that you’d like… find your book, and then you’d love it’. And then when I did, I got really annoyed at them even more because then I knew that they were right.

(Advisor 8)

Such passages give reason to explore age subgroups and also adjusting for confounding factors, such as spelling or reading disabilities in statistical analyses, that could have an effect on both reading engagement and also mental health. Additionally, it also highlights the need for measures to capture nuances between leisurely reading (self‐directed) for pleasure, and instructional reading, as many members noted that being ‘forced’ to read something reduced the enjoyment gained from the experience:

it makes me feel kind of annoyed. Like… I don’t enjoy it as much… even if it wasn’t gonna be joyful for me to read it, I’m still gonna.

(Advisor 9)

### Guiding users: DigiCAT tutorial paper

The YPAGs identified social media as a highly relevant and relatable focus due to its pervasive impact on young people's mental health. By selecting this topic, they ensured the project addressed an area of contemporary significance with direct relevance to the target user community. This choice emphasised the importance of investigating real‐world issues with actionable implications, guiding the research questions toward areas with practical resonance and impact.

The focus on social media also directly influenced the example tutorial in the publication introducing the tool to a user audience. This tutorial paper demonstrates the step‐by‐step application of propensity score analysis to explore how social media usage affects mental health outcomes.

We also developed guidance for tool users on incorporating LE expertise into their research, in consultation with young advisors, highlighting why this is a beneficial step for their projects. For example, we asked our advisors whether they believed young people should be involved in research about their mental health and, if so, why. Alongside unanimous agreement on the importance of including their perspectives, our YPAG advisors identified numerous benefits of youth involvement in mental health research. They expressed a desire to contribute meaningfully to research efforts, helping to interpret the data and identifying areas where interventions could be most impactful. Drawing upon their school experiences with research, which primarily involved responding to surveys and questionnaires, our advisors expressed frustration at the lack of clarity regarding the research purpose and how researchers would utilise the results. By involving young people in project planning, they could help shape more relevant research questions. For instance, as our advisors highlighted, they could provide input on survey response formats, such as deciding between open and closed questions and determining ‘which questions should have a tick box or which should have a free text box’. (Advisor 2). Furthermore, Advisor 2 noted that ‘if there is a clearer sense of what the research is going to be used for, why it's helping, I think then people might answer a lot more truthfully’, and therefore the results might be more useful. This valuable insight has been integrated into our tool tutorials and has influenced our own approach to incorporating LE expertise.

Crucially, being involved in mental health research can be an affirming experience. As Advisor 1 explained, ‘it would be beneficial because if the people filling out the form could see that it has been created by people the same age and who have had the same experiences then it might make them feel like they are actually being heard, and the questions are more relevant because they have been made specifically for them by similar people’. Being involved in mental health research could show individuals they are not alone in their experience. The impact of including LE collaborators extends to positive outcomes for those involved, aligning with existing research showing benefits like empowerment, a sense of purpose, acquisition of new skills and knowledge, and playing an active role in reshaping and improving mental health research and interventions (Worsley et al., 2022). This reciprocal relationship underscores the importance of valuing LEs for the enrichment of research endeavours and the empowerment of contributors. Engaging with those who bring distinctive LE perspectives enriches the research landscape.

In addition to shaping research priorities and tutorial examples, YPAG advisors informed the integration of user guidance within the DigiCAT tool itself. Drawing on their reflections about the importance of involving young people in mental health research and their frustrations with the lack of clarity and relevance they often experienced when participating in research, we developed instructions and guidance text that encourage DigiCAT users to incorporate LE expertise at key stages of their analyses. This guidance is embedded within the tool's built‐in tutorials, supporting users in generating research questions, selecting active ingredients for analysis, and interpreting findings. In this way, DigiCAT promotes a LE‐informed approach to research beyond this project, encouraging users to meaningfully engage with LE expertise in their own studies.

## DISSEMINATION

The advisors recognised the tool's potential to address crucial questions about their mental health needs. They suggested widespread distribution of the tool to key stakeholders concerned with young people, including headteachers and teachers, who possess valuable insights and data about them. Government agencies such as the Department for Education were identified as important targets, given their capacity to enact changes within schools.

To further enhance the reach and impact of DigiCAT, we plan to collaborate with Third Sector and mental health organisations actively addressing adolescent mental health challenges. While these might include people without requisite research training, they are beneficiaries of the research and important components in informing and designing research strategies and subsequent interventions. These organisations can play a pivotal role in raising awareness about the tool.

Researchers working in mental health fields, particularly those focusing on modifiable risk factors such as sleep quality, social media use, physical activity, and interpersonal relationships, will be priority dissemination targets. These researchers are a priority because young people identified these factors as important to their mental health and therefore the tool was designed and trialled with such research questions in mind. Importantly, DigiCAT addresses a common barrier faced by many researchers in this field, a lack of coding expertise, by providing an accessible platform and educational resources, including source code and step‐by‐step tutorials. The design of these resources was shaped by insights from the YPAGs, whose input helped ensure that the example tutorials demonstrated issues of particular relevance to young people, such as the impact of social media on mental health. These features also position DigiCAT as a valuable educational tool for early‐career researchers or those new to counterfactual methods, particularly those working on adolescent mental health topics identified by young advisors as priorities. In this way, YPAG contributions helped ensure DigiCAT is relevant to researchers addressing real‐world questions that matter to young people.

By targeting researchers who are already engaged with the topics prioritised by young people during the advisory sessions, DigiCAT aligns with existing efforts to address adolescent mental health challenges.

### Language and articulation

While discussing the dissemination of the tool with the advisors, there were queries about what language or languages it would be available in. This made us reflect on our existing desire to reach researchers globally. Following this we are in the process of translating the text of the tool and the accompanying instructions and tutorials into French and Spanish, selected as languages that are widely used.

## YOUTH‐REPORTED BARRIERS TO LE ENGAGEMENT

We asked the advisors what they thought might be possible challenges or barriers for young people engaging with research, as advisors. They identified several significant barriers rooted in social and emotional concerns, such as the difficulty of being open and honest due to anxieties about how they might be perceived or how their perspectives might be interpreted, which illuminate the complexities of involving adolescents in LE consultations.

Advisors frequently mentioned the difficulty of being open and honest, stemming from anxieties about stigma, perception, and judgement. Advisor 2 remarked:

some people talk a lot about mental health but don’t know much about it. There’s a lot of stigma about it, so some people don’t like talking about it.

This underscores the dual challenge of navigating both personal hesitations and broader societal stigma when discussing mental health issues.

There might be hesitancy from potential advisors in feeling that they can safely open up, and whether they could even name what it is they have experienced. This is further demonstrated by Advisor 2, who highlights the internal struggle they face articulating their experiences, stating:

if it doesn’t become a normalised thing people don’t even know how to explain anything.

This reflects the importance of fostering an environment where mental health discussions are encouraged and normalised, empowering young people to articulate their LEs more confidently.

Concerns about confidentiality and privacy were also prominent. As Advisor 1 noted:

maybe they would be worried that stuff that's obviously very individual and very private is going to be used as an example … if they might get named in research they could be nervous about that and they might dampen down, not tell the full truth about everything just because they're nervous about what people will do with that information, because it is really sensitive and private.

This highlights the necessity of clear communication about ethical practices, such as anonymisation and data protection, to alleviate fears of exposure and misuse of information. While all research projects and advisory group engagements seek explicit consent to name participants, this ethical practice and guarantee of non‐identifiability might not be immediately obvious to young people before they become advisors.

## ADDRESSING THE BARRIERS

Our approach to engaging LE advisors aimed to address these barriers through deliberate strategies. First, we emphasised recruiting advisors based on their general experiences as young people rather than explicitly targeting those with mental health challenges. This avoided placing undue pressure on advisors to disclose potentially sensitive or stigmatised personal experiences.

These insights underline the importance of transparency, sensitivity, and a supportive environment in facilitating meaningful youth engagement. Future research and practice should continue to explore innovative ways to address these barriers. For example, discussing mental health within the broader context of adolescence and overall well‐being can help normalise these conversations, making them feel more natural and less stigmatised. This approach allows young people to engage with mental health topics in a way that feels relevant and approachable, rather than isolating them as separate or sensitive issues. Providing training for both researchers and young advisors can further support this process; training researchers to engage sensitively and inclusively with young people and equipping young advisors with the language and confidence to articulate their LE. Additionally, research on co‐design highlights the value of dedicated team‐ and relationship‐building sessions that are not focused on research activities (Power et al., 2025). Creating spaces for informal connection can build trust and rapport, which are foundational for meaningful engagement and sustained collaboration. Leveraging peer‐support frameworks may also enhance young advisors' confidence and sense of belonging within advisory groups.

### General challenges and solutions

One key consideration in our research design was that young people were not the primary target users of the DigiCAT tool; instead, the tool was developed for use by mental health researchers. This distinction shaped how we incorporated YPAG input, focusing on areas where their LEs could meaningfully inform the project, rather than aspects requiring advanced methodological knowledge or technical design decisions.

The complexity of the tool's topic—focused on counterfactual analyses and statistical methods—meant that it was not feasible, sensible, or appropriate for every element of the tool to reflect young people's insights. Instead, the perspectives of YPAGs were channelled into two critical areas:

- *Tutorial paper guidance and research question generation examples*: The YPAGs provided valuable input into the types of research questions and case examples used to illustrate the tool's functionality. For instance, their discussions on the role of social media in adolescent mental health directly informed the design of the example tutorial included in the publication introducing DigiCAT, and in an empirical paper studying the effects of social media on adolescent mental health. This ensured that the demonstration of the tool's functionality was aligned with young people's LEs and research priorities, even though the tool itself was not designed for their direct use.
- *Dissemination strategies*: Young people's insights played a significant role in shaping how the tool was disseminated. Their suggestions highlighted the importance of reaching researchers working on adolescent mental health, as well as engaging educators and mental health organisations to maximise the tool's relevance and application to real‐world issues.

By focusing on these areas, we were able to incorporate young people's LEs into DigiCAT in ways that were meaningful and practical, without the pitfall of tokenistically involving them where it was not appropriate to do so (e.g., in the highly technical aspects of the tool design). This approach underscores the importance of working with LE experts while also acknowledging the limitations of indirect involvement in a highly technical research tool.

Nevertheless, this separation of target users and advisors presents certain limitations. While young people influenced specific elements of the project, their input did not extend to the design of the tool's technical functionalities or user interface. These aspects were shaped by the needs of researchers and required specialised methodological knowledge. Additionally, some components of the tool, such as its statistical methods and background processes, were not suitable for meaningful input from non‐specialised audiences.

Feedback on the tool's functionality and usability was gathered separately through a User Advisory Group (UAG), comprising researchers who represented the tool's intended end users. While the work of the UAG is not the focus of this paper, their insights were central to refining the prototype and ensuring it met the practical needs of mental health researchers.

Future projects might explore ways to increase the direct involvement of young people in supporting, or even co‐producing, tools of this complexity. While their role in DigiCAT focused on informing tutorial examples and dissemination, there is potential to broaden participation in future initiatives. For example, young advisors could be offered basic training in coding, such as creating simple Shiny Apps, to provide them with a deeper understanding of how digital tools are developed. Similarly, providing training in user experience (UX) research could enable young people to participate more actively in user testing, collecting feedback from other users, and informing iterative design decisions. These approaches could help shift youth involvement from consultation to more active participation in the development and refinement of research tools. Such involvement would require additional time, resources, and capacity building for both researchers and young people. However, it represents a promising avenue for embedding LE expertise more fully into digital mental health innovation.

While these limitations highlight areas for improvement, they also bring attention to the broader challenge of effectively engaging young people in discussions about complex and technical tools. Navigating the professional language of research presents a significant challenge for LE experts (Worsley et al., 2022). We addressed this challenge by conducting tailored sessions for young people, which avoided jargon and made use of interactive applications such as polling functions and Google Jamboard to make sessions more engaging. Our interdisciplinary team also facilitated better communication by ensuring we were in the habit of translating complex terms into terms understandable by those with different expertise. However, despite these efforts we still encountered notable challenges when discussing the digital tool itself with young people due to its technical nature and the fact that young people were not the target users.

Effective inclusion of LE experts necessitates strong relationship‐building, which often requires months or years. Ideally, LE experts would be integrated from the outset of a project and included in funding proposals. Achieving such integration in a holistic and meaningful way requires a significant shift in traditional research framework and timelines, as well as dedicated time and training for researchers in working collaboratively with LE experts.

Beyond the formal aspects of public engagement, relationships with communities must be fostered thoroughly, ensuring safety and appropriate safeguarding. Attention to the welfare of the advisors is paramount, with recognition of the emotional labour involved for the LE expert (Faulkner & Thompson, 2021). Building rapport and relationships with the individuals involved can help identify and acknowledge boundaries and potential points of limited engagement. Although our YPAGs all took place online, which was an inclusive, accessible, and convenient forum for discussion, this format may potentially impact the timeline required to establish these robust relationships.

While the vast majority of individuals engaging as LE experts in research are genuine and committed to contributing valuable insights, there may be instances where individuals attempt to exploit the system. For example, experts may provide inaccurate or fabricated information about their personal experiences, background, or perspectives, either intentionally or unintentionally, in order to receive compensation offered as part of the project. Screening procedures can help ensure that advisors are genuine and appropriate for the advisory role. More generally, building trust and rapport encourages open and honest participation and reduces the likelihood of deceptive behaviour. From our experience, we learnt the importance of robust verification processes, particularly in online recruitment. Future projects could benefit from advanced screening mechanisms, such as using unique participant codes or employing external verification platforms, to further ensure the authenticity and quality of advisors involved in virtual studies (Ridge et al., 2023; Sharma et al., 2024).

In our project, we do not believe we were impacted by imposter participants, partly due to the rapport we built up with advisors over multiple sessions and the intentionally small group sizes that enabled active participation from all members. Because our advisors were predominantly under 18, our correspondence with their parents or guardians may also have contributed to ensuring genuine participation. We believe that the respect shown to our advisors, reflected in the quality of our engagement, safeguarding measures and fair compensation, helped foster reciprocal respect for the project and our time.

Researchers should also provide clear instructions on expectations for engagement, including standards for honesty, respect, and professionalism. Recruitment can be enhanced by highlighting the benefit of research engagement for others and the potential impact on practice, policy and research projects (McEvoy et al., 2023). We asked our advisors how they would promote being part of advisory groups based on their experiences. They noted that they felt their opinions were valued and their voices heard, which could be a key element that could be highlighted in recruiting future advisors.

## LIMITATIONS

We acknowledge the importance of collecting demographic data as a critical tool for monitoring inclusivity and ensuring transparency in research. In our study, we prioritised sensitivity and equity by treating young people as collaborators rather than subjects, and therefore did not collect personal information such as gender or ethnicity. Instead of actively selecting advisors to ensure diversity, we advertised the opportunity broadly to maximise representation. This approach sought to respect the autonomy and privacy of participants, but we recognise that it limited our ability to evaluate the inclusivity of our sample, and how variations in social and demographic characteristics may impact their experiences. While this decision was guided by a desire to prioritise participant comfort, future research could adopt strategies such as offering optional, anonymous demographic surveys to balance these considerations.

All YPAG sessions were conducted online, which facilitated geographical inclusivity and accessibility for advisors both within and outside the UK. This approach allowed individuals from diverse locations to contribute, including Ireland, France, and Romania, with a majority from Scotland and England. However, we acknowledge that online engagement may have inadvertently excluded certain demographics, particularly those with limited access to reliable Internet, appropriate devices, or a private environment for participation. These limitations may have influenced the diversity of perspectives captured in the study. Future work should consider hybrid engagement methods to mitigate these barriers and ensure broader participation.

Given that the young people were not the end‐users of DigiCAT, other routes for integrating LE expertise might have been sought. While involving mental health researchers with LE would be a viable option, we chose to prioritise the emphasis on adolescent mental health, and invest in the development of the YPAGs. These groups contributed through consultation, shaping the examples within a tutorial paper used to introduce the tool and offering insights that informed dissemination strategies. Meanwhile, adult user groups and testing sessions provided feedback on how LE input influenced the tool in practice. Thus, while YPAG input shaped specific elements related to DigiCAT's presentation and communication, the embedded elements were trialled by users to ensure their practicality and effectiveness.

## IMPLICATIONS AND FUTURE DIRECTIONS

This study highlights the importance of incorporating LE into mental health research. DigiCAT offers a user‐friendly platform for exploring modifiable mental health risk factors, bridging the gap between advanced analysis and real‐world applicability. Future research could explore more direct ways to involve young people in digital tool development, including youth‐specific modules or simplified interfaces. Providing basic training in areas such as coding, for example, creating simple Shiny Apps, or UX research, including facilitating user testing and gathering feedback, could enable young advisors to contribute more actively to design and development processes. These approaches would require additional time and resources but offer promising opportunities for deeper engagement of young experts by experience. Moreover, greater attention to inclusivity in recruitment and the use of demographic data could enhance the representativeness and equity of future projects. Overall, DigiCAT demonstrates how integrating LE expertise can lead to more effective and relevant research tools, setting a precedent for future digital initiatives in adolescent mental health.

## CONCLUSION

In conclusion, our experiences highlight the substantial benefits of the inclusion of LE experts in research, even when they are not the direct end‐users of the resulting tools. This project demonstrated the significant value of integrating LE expertise including generating pertinent research questions, inputting on tutorial materials, interpreting and framing findings, and informing dissemination strategies. As part of this, we embedded guidance within DigiCAT's built‐in tutorials to encourage users to incorporate LE expertise at key stages of their analyses, ensuring the tool supports a research process that is both rigorous and grounded in the lived realities of young people. The young advisors provided nuanced first‐hand insights into adolescent mental health, identifying key factors such as social media usage and reading habits, which informed both empirical analyses and tutorial examples. They also highlighted barriers to inclusion, such as stigma, privacy concerns, and the challenges of navigating professional research language, while offering invaluable solutions and dissemination strategies that extend beyond academia.

## AUTHOR CONTRIBUTIONS

**Marie Allitt**: Formal analysis; funding acquisition; investigation; methodology; project administration; writing—original draft; writing—review and editing. **Helen Wright**: Data curation; formal analysis; funding acquisition; investigation; methodology; resources; writing—original draft; writing—review and editing. **Aja Murray**: Conceptualization; formal analysis; funding acquisition; investigation; methodology; project administration; supervision; writing—original draft; writing—review and editing. **Ingrid Obsuth**: Funding acquisition; investigation; methodology; project administration; resources; supervision; writing—original draft; writing—review and editing.

## CONFLICT OF INTEREST STATEMENT

The authors declare no conflicts of interest.

## ETHICAL CONSIDERATIONS

Ethical approval was not required for this publication, as it describes the involvement of young people in an advisory capacity to inform the development of an app. This involvement did not constitute participation in a research study and did not involve the collection of research data. Therefore, informed consent and formal ethical review were not applicable.

## Data Availability

Data sharing is not applicable to this article as no datasets were generated or analysed during the current study.
